# Phase-Specific Assessment of Corrosion Susceptibility in Inconel 625 and SA508 Low-Alloy Steel Under Molten Chloride Conditions

**DOI:** 10.3390/ma19143139

**Published:** 2026-07-22

**Authors:** Seongwon Ham, Hyung-Ha Jin, Chaewon Kim, Jinsuo Zhang, Sangtae Kim

**Affiliations:** 1 Department of Nuclear Engineering, Hanyang University, Seoul 04763, Republic of Korea; 2 Materials Safety Technology Research Division, Korea Atomic Energy Research Institute, Daejeon 34057, Republic of Korea; 3 Nuclear Engineering Program, Department of Mechanical Engineering, Virginia Polytechnic Institute and State University, Blacksburg, VA 24061, USA; 4 Department of Materials Science and Engineering, Hanyang University, Seoul 04763, Republic of Korea

**Keywords:** molten salt corrosion, Ni-based alloy, computational corrosion susceptibility prediction

## Abstract

Nickel-based alloys are promising structural materials for molten salt systems; however, secondary-phase formation during long-term high-temperature exposure may introduce local corrosion susceptibility because secondary phases have compositions and redox stabilities distinct from the matrix. Here, we combine CALculation of PHAse Diagrams (CALPHAD)-based phase prediction with redox thermodynamic analysis to assess phase-specific corrosion susceptibility in Inconel 625 (IN625) and SA508 low-alloy steel under molten chloride conditions. Equilibrium phase constitutions at 1000 K were predicted using Thermo-Calc, and redox equilibrium potentials were calculated for representative-phase dissolution reactions of major metallic elements in each phase. The dominant α and γ phases in SA508 exhibited similar Fe-ionization potentials of −1.728 and −1.768 V vs. Cl2/Cl^−^, respectively. In IN625, the γ matrix exhibited a Cr-ionization potential of −1.964 V vs. Cl2/Cl^−^, whereas the P phase showed the most negative potential of −2.132 V vs. Cl2/Cl^−^, 0.168 V more negative than the matrix, identifying the P phase as the primary local thermodynamic weak point. These results show that phase-specific metal-ionization susceptibility cannot be inferred solely from nominal alloy composition or matrix behavior. The proposed framework provides a thermodynamic screening approach for identifying susceptible secondary phases in multicomponent alloys under molten-salt conditions.

## 1. Introduction

Ni-based alloys have been widely considered as candidate structural materials for molten salt reactors owing to their corrosion resistance in high-temperature molten salts [1,2,3,4,5,6,7,8,9]. Their Ni-rich γ matrix generally exhibits a low metal-ionization tendency in chloride and fluoride salts, while alloying elements such as Cr, Mo, and Nb improve high-temperature strength, creep resistance, and oxidation resistance [10,11]. During prolonged exposure at reactor operating temperatures, however, these elements can partition into secondary phases, including γ″ precipitates, carbides, and topologically close-packed (TCP) phases [10,12,13,14,15,16,17,18]. Because these phases have compositions and thermodynamic stabilities distinct from the matrix, their corrosion responses may also differ from that of the surrounding γ matrix. Thus, understanding the role of secondary phases is important for evaluating corrosion-relevant heterogeneity in Ni-based alloys under molten salt reactor conditions.

Evidence for phase-dependent corrosion effects has been reported primarily in aqueous corrosion studies of Ni-based alloys [19,20,21,22,23,24]. In Ni-based superalloys such as Inconel 718, precipitated phases including Laves phases, carbides, γ″, and δ phases can form during thermal exposure, producing local microstructural and electrochemical heterogeneities [19,20]. In chloride-containing aqueous solutions, localized corrosion of Inconel 718 has been observed preferentially at interfaces between the austenitic matrix and metal carbides, suggesting that secondary-phase/matrix heterogeneity can provide initiation sites for localized attack [21]. In a Ni–Cr–Mo superalloy, increasing the fraction of a P-type TCP phase in a chloride-rich aqueous solution shifted the corrosion potential negatively and increased the anodic current density, with localized attack proceeding through selective dissolution of the P phase [22]. These studies demonstrate that individual secondary phases can exhibit corrosion responses distinct from those of the surrounding matrix [21,22,23]. Although the interfacial reactions and corrosion mechanisms in aqueous solutions differ from those in high-temperature molten salts, these observations establish that phase-specific composition and stability can generate localized electrochemical heterogeneity within Ni-based alloys.

Although phase-specific corrosion data for Ni-based alloys remain limited in molten salts compared with aqueous environments, recent experimental and modeling studies have clarified the roles of selective elemental dissolution, near-surface compositional redistribution, and phase evolution in molten-chloride corrosion. Experiments on Fe- and Ni-based structural alloys have revealed selective dissolution and substantial changes in near-surface alloy chemistry, although the responses of individual secondary phases were not separately resolved [25]. Building on a CALPHAD-based thermodynamic–kinetic framework originally developed to predict compositional and phase evolution in multicomponent alloys [26], subsequent models reproduced experimentally observed Cr depletion together with Mo and W enrichment in binary Ni–Cr and commercial Ni-based alloys exposed to molten KCl–MgCl_2_ [27,28]. These studies further showed that elemental activity and solid-state diffusion are more informative than nominal alloy composition alone for describing corrosion-induced compositional evolution and improving long-term corrosion predictions [27,28,29]. However, because these approaches primarily predict alloy-scale compositional and phase evolution, they do not directly identify the intrinsic metal-ionization susceptibility of each pre-existing matrix and secondary phase. Consequently, whether an individual secondary phase is thermodynamically more susceptible to metal ionization than the surrounding matrix under the same molten-salt condition remains unresolved.

In this study, we combine CALculation of PHAse Diagrams (CALPHAD)-based phase prediction with redox thermodynamic assessment to compare phase-specific susceptibility to metal ionization in multiphase structural alloys under molten chloride conditions. Inconel 625 (IN625) was selected as a representative Ni-based superalloy containing compositionally distinct secondary phases [30], whereas SA508 low-alloy steel was included as a compositionally simpler Fe-based reference alloy. CALPHAD calculations provided the phase fractions and compositions of the matrix and secondary phases [30,31], which were used to construct representative atomic structures and calculate redox equilibrium potentials for the ionization of their constituent metallic elements [32]. This analysis identifies the most favorable metal-ionization reaction within each phase and compares the intrinsic redox stability of the matrix and secondary phases. The calculated potentials are interpreted as phase-specific thermodynamic descriptors rather than direct predictions of corrosion rate or electrochemical mixed potential.

## 2. Methods

### 2.1. Calphad-Based Phase Prediction

**Figure 1:**
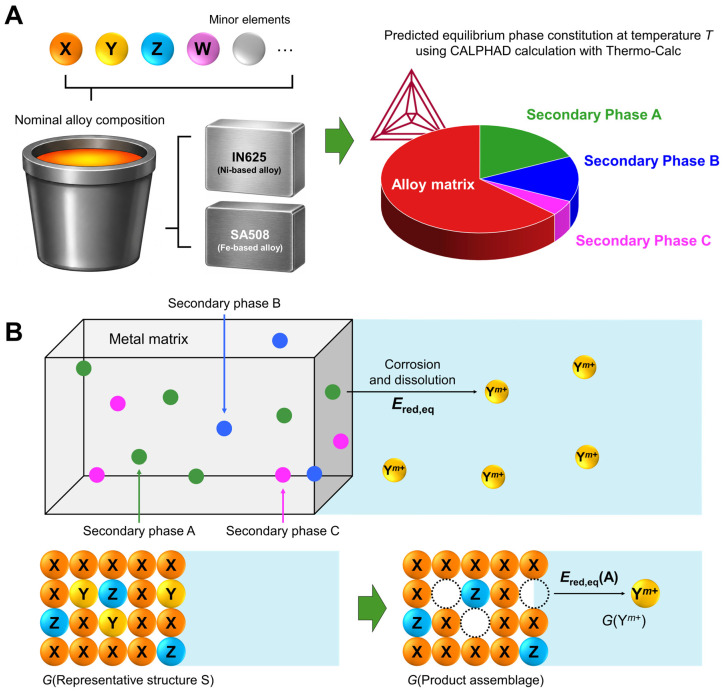
**Schematic illustration of the computational workflow used for phase-specific corrosion thermodynamic assessment.** (**A**) CALPHAD calculations using Thermo-Calc were performed based on the nominal compositions of SA508 and IN625 to predict the equilibrium phase constitution, including stable phases, phase fractions, and phase-specific compositions at temperature *T*. (**B**) Representative atomic structures were constructed for the alloy matrix and secondary phases. Each representative structure was treated as a compound-like thermodynamic unit, and its stability was compared with product assemblages consisting of dissolved metal ions and the reference states of the remaining constituent elements. The resulting dissolution Gibbs free energy was normalized by the number of dissolving metal atoms and converted into the redox equilibrium potential, *E*_red,eq_, to compare the thermodynamic tendency for metal ionization among different phases.

The high-temperature equilibrium phase constitutions of IN625 and SA508 were predicted using Thermo-Calc 2025b [31]. The TCNI13 Ni-based alloy database was used for IN625, whereas the TCFE14 Fe-based alloy database was used for SA508. The nominal compositions of IN625 and SA508 used in the calculations are listed in Table 1. The compositions listed in Table 1 were selected as representative nominal compositions within the published composition limits for IN625 (UNS N06625) and SA508 Grade 3 Class 1 [32,33]. They were used as computational input compositions and do not represent measured compositions of specific material heats. All equilibrium calculations were performed at a pressure of 1 atm. The CALPHAD calculations were conducted in two steps. First, temperature-dependent equilibrium calculations were performed over the range of 400–1000 °C to evaluate the formation and evolution of secondary phases in each alloy. These calculations were used to determine how the phase fractions changed across the operating-temperature range relevant to molten salt reactor environments. Second, separate isothermal equilibrium calculations were performed at 1000 K to obtain the phase-specific thermodynamic information required for the subsequent corrosion assessment. From these calculations, the stable phases at 1000 K, their phase fractions, and their elemental compositions were extracted (Figure 1A). The obtained phase compositions were then used as inputs for constructing representative atomic structures for the alloy matrix and secondary phases. Because IN625 and SA508 are multicomponent commercial alloys, their phase constitutions were calculated using the complete nominal compositions listed in Table 1 rather than simplified ternary subsystems.

### 2.2. Construction of Representative Phase Structures

Representative atomic structures were constructed for each alloy matrix and secondary phase using the phase compositions predicted by CALPHAD at 1000 K. The matrix phases were generated from their corresponding crystal structures: the face-centered cubic (FCC) structure was used for the γ matrix, and the body-centered cubic (BCC) structure was used for the α matrix. For the secondary phases, phase-specific parent structures were used instead of elemental crystal structures. The γ″ phase was constructed based on the Ni_3_Nb-type structure, whereas the carbide phases in both IN625 and SA508 were constructed based on the M_23_C_6_ structure. The P phase was constructed using an ordered Ni–Cr–Mo P-phase structure as the parent structure. The elemental occupations of the parent structures were then modified to reproduce the CALPHAD-predicted phase compositions at 1000 K as closely as possible. For matrix phases and P phase, alloying elements were substituted over the metallic sites according to the target phase compositions. For M_23_C_6_ carbides, carbon sites were kept fixed, and only the metal sublattice was substituted with alloying elements such as Cr, Mo, Fe, and Ni. For the γ″ phase, the Ni and Nb sublattices were distinguished during substitution so that the characteristic Ni_3_Nb-type site occupation was retained. Because CALPHAD-predicted compositions are continuous values, the number of substituted atoms was rounded to integer values while minimizing the deviation from the target composition within the finite supercell size. To account for configurational effects associated with atomic site occupation, 200 random atomic configurations were generated for each phase at a fixed composition and atom number. Each configuration was structurally relaxed, and its total energy was evaluated. Because the configurations generated for a given phase had the same composition and number of atoms, the configuration with the lowest total energy was selected as the representative structure of that phase. The selected representative structures were subsequently used for representative-phase redox thermodynamic calculations. Structural relaxation and energy calculations were performed using Matlantis. The generalized gradient approximation (GGA)-level universal PreFerred Potential (PFP) implemented in Matlantis v8.0.0 was used for all calculations [34]. Vibrational contributions at 1000 K were included in the Gibbs free energies used for evaluating phase-specific dissolution reactions.

### 2.3. Representative-Phase Dissolution Model and Redox Equilibrium Potential Calculation

To evaluate the thermodynamic tendency of each alloy matrix or secondary phase to release metallic elements into molten chloride, each representative structure was treated as a compound-like thermodynamic unit. This approach follows the phase-stability framework used in our previous molten-salt corrosion assessment [35], in which the stability of a compound phase is compared with that of possible product assemblages consisting of dissolved metal ions and the reference states of the remaining constituent elements. Therefore, the calculated redox equilibrium potential represents the potential at which a representative structure becomes thermodynamically unstable with respect to metal-ion formation. For each representative structure S, the dissolution of a metallic element M was expressed as (Figure 1B):

(1) $$S\to N_{M}M^{n+}+\sum_{i\neq M}N_{i}X_{i}^{ref}+N_{M}ne^{-}$$

where *N*_M_ is the number of atoms of element M in the representative phase, M*^n^*^+^ is the corresponding dissolved metal ion in the molten salt, *n* is the oxidation state of the dissolved ion, and X_i_^ref^ denotes the reference state of each remaining constituent element X_i_. For example, when Cr dissolution is evaluated, Cr atoms in the representative structure are converted into dissolved Cr*^n^*^+^ ions, whereas the other constituent elements are retained in their reference states. This reaction should be interpreted as a thermodynamic construction for comparing the stability of a representative structure against metal-ion formation, rather than as a mechanistic description of simultaneous dissolution of all M atoms from the phase. The dissolution Gibbs free energy was calculated as the difference between the product assemblage and the initial representative structure and normalized by the number of M atoms in the representative structure:

(2) $$\Delta G_{diss}^{S}\left(M|M^{n+}\right)=\frac{N_{M}\mu \left(M^{n+}\right)+\sum_{i\neq M}N_{i}\mu (X_{i}^{ref})-G\left(S\right)}{N_{M}}$$

where *G*(S) is the Gibbs free energy of the representative structure, *μ*(M*^n^*^+^) is the chemical potential of the dissolved metal ion in the molten salt, and *μ*(X_i_^ref^) is the chemical potential of each remaining constituent element in its reference state. This normalization allows the dissolution tendency to be compared on a per M atom basis among different representative structures with different compositions and numbers of atoms. Only metallic elements were considered as possible ionized species. Non-metallic elements such as carbon in carbide phases were retained as non-ionized constituent elements in the product assemblage and were not evaluated as dissolved ions.

The structural Gibbs free energy *G*(S) was evaluated from the relaxed representative structure obtained using Matlantis, with vibrational contributions included at 1000 K. The chemical potential of the dissolved metal ion was calculated following the molten-salt phase-stability framework reported in our previous work [35]. For each metallic element M, a pure-element bulk structure containing approximately 100–150 atoms was constructed and structurally relaxed using the same Matlantis/PFP calculation framework employed for the representative phase structures. The Gibbs free energy of the relaxed bulk structure, *G*(M_bulk_), was divided by the total number of atoms, *N*_M_, to obtain the per-atom bulk reference chemical potential, *μ*(M_bulk_) = *G*(M_bulk_)/*N*_M_. The standard Gibbs free energy of formation of the corresponding metal chloride, MCl*_n_*, at 1000 K, Δ*G*_f_º (MCl*_n_*), was obtained from HSC Chemistry version 10.0.8.0. No explicit bulk carrier-salt composition was specified because HSC Chemistry was used only to obtain the standard thermochemical data for MCl*_n_*, rather than to perform a multicomponent molten-salt solution calculation. The standard chemical potential of the metal ion in the molten salt was then defined as:

(3) $$\mu {}^{\circ}\left(M^{n+}\right)=\mu {}^{\circ}\left(M_{bulk}\right)+\Delta G_{f}{}^{\circ}(MCl_{n})$$

The chemical potential of the metal ion under the dilute molten chloride condition was calculated by applying the Nernst equation:

(4) $$\mu \left(M^{n+}\right)=\mu {}^{\circ}\left(M^{n+}\right)+RTln[a(M^{n+})]$$

where *R* is the gas constant, *T* is the temperature, and *a*(M*^n^*^+^) is the activity of the dissolved metal ion in the molten salt. In this study, ideal dilute behavior was assumed, and the activity of all dissolved metal ions was set to 10^−6^. Solvent-specific activity coefficients and complexation effects were not considered. The dissolution Gibbs free energy was converted into the redox equilibrium potential using the oxidation state of the dissolved metal ion and the Faraday constant:

(5) $$E_{red,eq}^{S}(M/M^{n+})=-\Delta G_{diss}^{S}(M/M^{n+})/nF$$

where *F* is the Faraday constant. The metal chloride species and oxidation states used to define M*^n^*^+^ were selected following the same convention as in the previous molten-salt phase-stability framework [35]. For each representative structure, separate metal-ionization reactions were evaluated for all metallic elements present in the structure. The redox equilibrium potential, *E*^S^_red,eq_, was calculated for each candidate element under the same temperature and dissolved-ion activity conditions. The element yielding the most negative *E*^S^_red,eq_ was identified as the thermodynamically preferred ionizing element for that phase, and the corresponding potential was used to represent the highest metal-ionization susceptibility of the structure. This approach follows the conventional thermodynamic ranking used in molten-salt corrosion assessments, in which the relative redox potentials of constituent metallic elements are compared to identify the element most susceptible to oxidation and dissolution [6]. This selection provides a thermodynamic ranking of possible ionization reactions and does not imply that only the selected element can dissolve under actual corrosion conditions. All redox equilibrium potentials were reported with respect to the Cl_2_/Cl^−^ reference electrode. The calculated potentials were used as thermodynamic descriptors for comparing the tendency of specific metallic elements in each phase to dissolve into the molten salt, rather than as direct predictions of corrosion rate or mixed corrosion potential.

## 3. Results and Discussion

### 3.1. Calphad-Derived Phase Constitution and Representative Phase Structures

**Figure 2:**
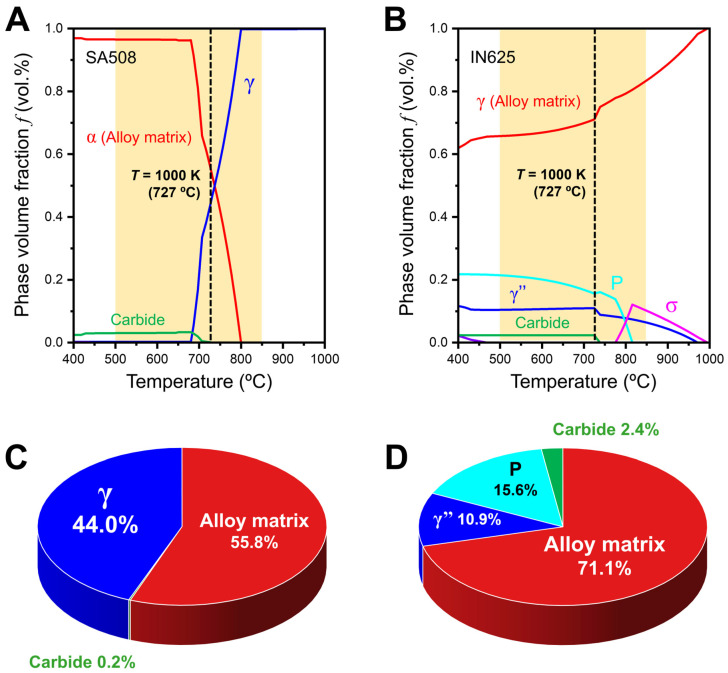
**CALPHAD-predicted equilibrium phase constitutions calculated using the complete nominal multicomponent compositions of SA508 and IN625.** (**A**,**B**) Temperature-dependent phase volume fractions of SA508 and IN625 over the range of 400–1000 °C. The highlighted region indicates the operating-temperature window commonly considered for molten salt reactor systems. (**C**,**D**) Equilibrium phase constitutions of SA508 and IN625 predicted at 1000 K.

CALPHAD calculations were first performed to predict the high-temperature equilibrium phase constitutions of SA508 and IN625. The purpose of this step was to identify which alloy matrix and secondary phases can exist under high-temperature conditions relevant to molten salt systems and to obtain phase-specific compositions for constructing representative atomic structures. Figure 2A,B show the temperature-dependent phase volume fractions of SA508 and IN625 over the range of 400–1000 °C, with the highlighted region indicating the operating-temperature window commonly considered for molten salt reactor systems. Figure 2C,D summarize the equilibrium phase constitutions at 1000 K. This temperature was selected as the reference temperature for the subsequent representative-structure construction and redox thermodynamic assessment.

For SA508, the Fe-rich α alloy matrix was predicted to be the dominant phase at lower temperatures, accompanied by a small amount of carbide phase. As the temperature increased, the volume fraction of the γ phase increased progressively, whereas that of the α alloy matrix decreased. The carbide phase remained a minor phase throughout the calculated temperature range and decreased to a very small fraction at high temperatures. At 1000 K, SA508 consisted mainly of the α alloy matrix and γ phase, with volume fractions of 55.8 and 44.0 vol.%, respectively, while the carbide phase accounted for only 0.2 vol.% (Figure 2C). These results indicate that the high-temperature equilibrium phase constitution of SA508 consists predominantly of Fe-rich matrix phases, with carbide present only as a minor secondary phase.

In contrast, IN625 exhibited a Ni-rich γ alloy matrix as the dominant phase throughout the temperature range considered, together with substantial fractions of secondary phases. At lower temperatures, the P and γ″ phases occupied relatively large fractions. The BCC phase, represented by the purple line in Figure 2B, was predicted to be stable only in the low-temperature range and was no longer present above 500 °C. With increasing temperature, the fractions of these phases decreased, while an additional σ phase was stabilized in the higher-temperature range. At 1000 K, the equilibrium phase constitution of IN625 consisted of 71.1 vol.% γ alloy matrix, 15.6 vol.% P phase, 10.9 vol.% γ″ phase, and 2.4 vol.% carbide phase (Figure 2D). Therefore, unlike SA508, which consisted mainly of Fe-rich matrix phases with only a trace amount of carbide, IN625 cannot be treated simply as a single Ni-rich γ matrix but should be considered as a multiphase alloy containing secondary phases with non-negligible phase fractions. This contrast suggests that corrosion-relevant heterogeneity should be assessed not only in terms of phase fractions but also in terms of phase-specific compositions that define the local dissolution environments.

**Figure 3:**
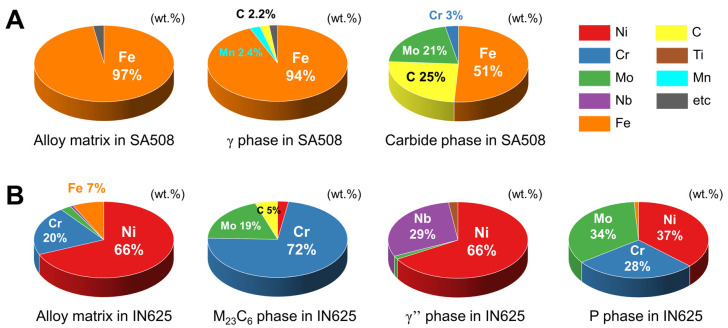
**CALPHAD-predicted phase-specific elemental compositions at 1000 K.** (**A**) Elemental compositions of the alloy matrix, γ phase, and carbide phase in SA508. (**B**) Elemental compositions of the γ alloy matrix, carbide, γ″ phase, and P phase in IN625.

Figure 3 shows the phase-specific elemental compositions of the alloy matrices and secondary phases predicted at 1000 K. For SA508, the **α** alloy matrix was predicted to be an Fe-rich phase containing approximately 97 wt.% Fe. The γ phase was also Fe-rich, containing approximately 94 wt.% Fe, 2.4 wt.% Mn, and 2.2 wt.% C. Thus, the two major phases in SA508 were compositionally similar, and both were dominated by Fe. In contrast, the carbide phase exhibited a composition clearly distinct from those of the matrix phases, consisting of approximately 51 wt.% Fe, 25 wt.% C, 21 wt.% Mo, and 3 wt.% Cr. Although the carbide phase accounted for only 0.2 vol.% at 1000 K, its enrichment in Cr, Mo, and C indicates that a local chemical environment distinct from the Fe-rich matrix can form within SA508. However, because of its very small volume fraction, the carbide phase is expected to have a limited contribution to the overall alloy-level thermodynamic response of SA508.

For IN625, the γ alloy matrix consisted mainly of Ni, with approximately 66 wt.% Ni, 20 wt.% Cr, and 7 wt.% Fe, reflecting the typical composition of a Ni-rich matrix. However, the secondary phases exhibited compositions markedly different from those of the γ matrix. The carbide phase was strongly enriched in Cr and Mo and contained only a minor amount of Ni, with approximately 72 wt.% Cr, 19 wt.% Mo, and 5 wt.% C. The γ″ phase was identified as a Ni–Nb-based intermetallic phase containing approximately 66 wt.% Ni and 29 wt.% Nb, whereas the P phase was a Cr–Mo-rich intermetallic phase containing approximately 37 wt.% Ni, 34 wt.% Mo, and 28 wt.% Cr. These results show that the compositions of the secondary phases differ substantially from the nominal IN625 composition, with Cr and Mo preferentially concentrated in the carbide and P phases and Nb concentrated in the γ″ phase. The CALPHAD-predicted phase compositions are qualitatively consistent with previously reported phase chemistry. The Ni-rich γ matrix and Nb-enriched γ″ phase agree with the elemental partitioning reported for Alloy 625 [15], whereas the Cr-rich carbide and Cr–Mo-rich P phase follow the established compositional characteristics of these secondary phases [11,36]. The Fe-rich α and γ phases predicted for SA508 are also consistent with the Fe-balanced composition of this low-alloy steel. These comparisons provide a qualitative consistency check rather than direct validation for a specific material heat.

Phase-dependent compositional partitioning can influence molten chloride corrosion susceptibility because secondary phases provide local chemical environments distinct from those of the γ matrix. Therefore, secondary phases with compositions distinct from the matrix may exhibit different dissolution behavior in molten chloride. In IN625, the P phase combines a non-negligible phase fraction with strong Cr and Mo enrichment, while the carbide phase represents a smaller but highly Cr-enriched secondary phase. By contrast, the γ″ phase is mainly composed of Ni and Nb and contains little Cr, suggesting that its dissolution behavior may differ from those of the Cr-containing phases. These results indicate that phase fraction and phase-specific composition must be considered together when assessing corrosion-relevant heterogeneity in multiphase alloys. Therefore, the CALPHAD-derived phase compositions shown in Figure 3 provide the basis for constructing representative atomic structures and evaluating phase-specific redox equilibrium potentials for representative-phase dissolution reactions.

### 3.2. Phase-Specific Redox Equilibrium Potentials of Alloy Matrix and Secondary Phases

**Figure 4:**
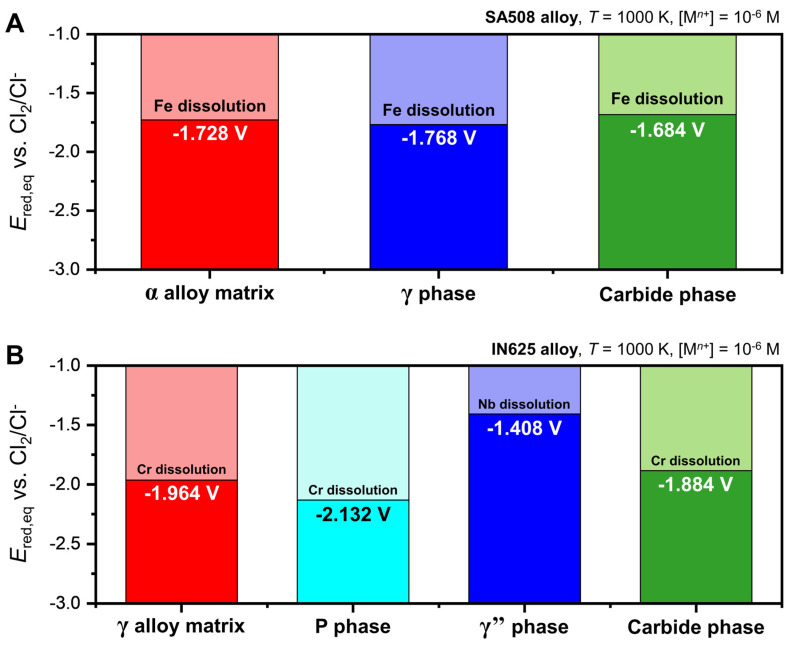
**Phase-specific redox equilibrium potentials of the alloy matrix and secondary phases at 1000 K under a dissolved metal-ion activity of 10^−6^.** (**A**) Redox equilibrium potentials calculated for the α alloy matrix, γ phase, and carbide phase in SA508. (**B**) Redox equilibrium potentials calculated for the γ alloy matrix, P phase, γ″ phase, and carbide phase in IN625. All potentials are reported with respect to the Cl_2_/Cl^−^ reference electrode.

Figure 4 summarizes the resulting phase-specific redox equilibrium potentials for SA508 and IN625. In this work, *E*_red,eq_ was used as a thermodynamic descriptor for comparing the tendency of a metallic element in a given phase to dissolve into molten chloride as a metal ion. A more negative *E*_red,eq_ indicates a greater thermodynamic tendency for metal ionization under the same dilute molten chloride condition. For each phase, Figure 4 shows the most negative *E*_red,eq_ value selected from the set of dissolution reactions considered for that phase. The reported value therefore provides a phase-specific measure of the highest thermodynamic tendency for metal ionization under the present calculation condition.

For SA508, Fe dissolution was identified as the most susceptible dissolution reaction for the α alloy matrix, γ phase, and carbide phase (Figure 4A). The calculated *E*_red,eq_ values for Fe dissolution from the α alloy matrix, γ phase, and carbide phase were −1.728, −1.768, and −1.684 V vs. Cl_2_/Cl^−^, respectively. The full spread among these values is approximately 84 mV, and the two dominant phases, the α alloy matrix and γ phase, differ by only approximately 40 mV. This indicates that the dominant phases in SA508 have broadly comparable Fe-dissolution tendencies under the present calculation condition. The CALPHAD-derived compositions shown in Figure 3A provide a compositional basis for this result, as the α alloy matrix and γ phase were both dominated by Fe. Therefore, although SA508 forms more than one phase at 1000 K, its major phases show similar Fe-rich compositions and comparable Fe-dissolution potentials. The carbide phase also exhibited Fe dissolution as its most susceptible reaction; however, its *E*_red,eq_ value was the most positive among the SA508 phases. This indicates that Fe ionization from the carbide phase is thermodynamically less favorable than that from the α alloy matrix and γ phase under the present calculation condition. Therefore, the carbide phase could, in principle, represent a less susceptible local phase in SA508. Nevertheless, because the carbide phase accounts for only approximately 0.2 vol.% of SA508 at 1000 K (Figure 2C), such a beneficial contribution is expected to be negligible at the alloy scale. Instead, the thermodynamic dissolution susceptibility of SA508 is expected to be dominated primarily by Fe dissolution from the α alloy matrix and γ phase. In this sense, SA508 serves as a useful Fe-based reference alloy in which the phase-specific redox response is relatively homogeneous among the dominant phases.

The behavior of IN625 is markedly different from that of SA508. For the γ alloy matrix of IN625, Cr was identified as the most thermodynamically susceptible dissolving element, with an *E*_red,eq_ value of −1.964 V vs. Cl_2_/Cl^−^ (Figure 4B). This result indicates that, despite the Ni-rich composition of the γ matrix, Cr is the metallic element most prone to ionization from this phase under the present calculation condition [8,30,37]. Among the secondary phases in IN625, the P phase exhibited the most pronounced thermodynamic susceptibility, showing Cr dissolution with the most negative *E*_red,eq_ value of −2.132 V vs. Cl_2_/Cl^−^. This value is approximately 0.168 V more negative than that of the γ matrix, indicating that the Cr-dissolution reaction defined for the P-phase representative structure is thermodynamically more favorable than that defined for the γ-matrix representative structure. Because the P phase exhibits the most negative Cr-dissolution potential and accounts for a substantial phase fraction of approximately 15.6 vol.% at 1000 K, it may increase the overall thermodynamic dissolution susceptibility of IN625 in molten chloride. This result is notable because the P phase contains a substantial amount of Mo, but within the set of representative-phase dissolution reactions considered in the present calculation, Mo dissolution was less thermodynamically favorable than Cr dissolution [9,38]. Thus, the high thermodynamic susceptibility of the P phase cannot be explained simply by elemental enrichment but instead reflects the phase-specific thermodynamic stability of the representative structure relative to Cr-ion formation.

In contrast, the carbide phase showed a Cr-dissolution potential of −1.884 V vs. Cl_2_/Cl^−^, which is approximately 0.080 V more positive than that of the γ matrix. This positive shift indicates that the Cr-rich carbide phase is not thermodynamically more susceptible to Cr ionization than the γ matrix under the present calculation condition, despite its strong Cr enrichment and distinct local composition [39]. The γ″ phase exhibited a distinct dissolution response from the Cr-dissolving γ matrix, P phase, and carbide phase. In this phase, Nb dissolution was identified as the most susceptible dissolution reaction, with an *E*_red,eq_ value of −1.408 V vs. Cl_2_/Cl^−^. This value is substantially more positive than the Cr-dissolution potentials of the γ matrix, P phase, and carbide phase, indicating that the γ″ phase has a lower thermodynamic tendency for metal ionization. This result can be understood from the phase-specific composition shown in Figure 3B, where the γ″ phase is mainly composed of Ni and Nb and contains little Cr. Accordingly, the γ″ phase can be interpreted as a Nb-controlled dissolution response rather than a Cr-controlled one. This change in the dominant dissolving element explains the relatively positive *E*_red,eq_ value of the γ″ phase, because Nb ionization was less thermodynamically favorable than Cr ionization within the set of dissolution reactions considered in the present calculation. Therefore, compared with the P phase, which exhibits the most negative Cr-dissolution potential among the IN625 phases, the γ″ phase is unlikely to act as the primary thermodynamic weak point in IN625.

These results demonstrate that the molten chloride corrosion susceptibility of IN625 cannot be inferred solely from the behavior of the Ni-rich γ matrix. Although the Ni-rich γ matrix exhibits a relatively low thermodynamic tendency for metal ionization, the equilibrium phase constitution of IN625 includes secondary phases whose local compositions and redox responses differ substantially from those of the matrix. Therefore, the corrosion susceptibility of IN625 cannot be assessed from the Ni-rich matrix alone but must account for the phase-specific dissolution tendencies of secondary phases such as the P phase, carbide phase, and γ″ phase. In the present calculation, the P phase was identified as the most thermodynamically susceptible phase for Cr dissolution, whereas the carbide phase showed a Cr-dissolution potential slightly more positive than that of the γ matrix despite its strong Cr enrichment. In addition, the γ″ phase exhibited a Nb-controlled dissolution response with a substantially more positive *E*_red,eq_ value. These results indicate that secondary phases in IN625 do not contribute uniformly to corrosion susceptibility; instead, their effects depend on the phase-specific redox stability of the dissolving element.

The comparison between SA508 and IN625 further highlights the value of phase-resolved redox thermodynamic assessment. In SA508, the major phases are compositionally similar Fe-rich phases, and their Fe-dissolution potentials are also similar. As a result, the nominal alloy composition can capture the dominant Fe-dissolution behavior as a first approximation. In IN625, however, the nominal Ni-based composition masks the presence of secondary phases whose redox responses differ markedly from that of the γ matrix. Therefore, a nominal-composition-based or pure-element-based assessment would fail to identify the P phase as a local thermodynamic weak point, while also missing the distinct but less susceptible behavior of the carbide and γ″ phases. The qualitative trends predicted in this study are consistent with previous observations of preferential Cr depletion in Ni-based alloys exposed to molten chloride salts [8,9,30,37,39] and selective dissolution associated with P-type TCP phases in chloride-containing aqueous environments [21,22]. Although aqueous chloride solutions and high-temperature molten chlorides involve different corrosion mechanisms, these studies support the broader conclusion that phase-specific composition and stability can produce different local corrosion susceptibilities.

The present framework, which combines CALPHAD-predicted phase constitution with representative-structure-based redox thermodynamics, provides a means to identify both the phases and metallic elements that are thermodynamically susceptible to dissolution in molten chloride. This approach can serve as a thermodynamic screening tool for multiphase alloys, particularly when secondary phases are expected to form or persist under high-temperature service or heat-treatment conditions. For IN625 and related Ni-based alloys, suppressing or controlling the formation of the P phase may be important for reducing local thermodynamic dissolution susceptibility because this phase exhibits the most negative Cr-dissolution potential among the phases considered in this work. Although the phases are electrically connected in an actual multiphase alloy, the independently calculated phase-specific *E*_red,eq_ values can provide a first-order thermodynamic ranking at the initial stage of corrosion, before substantial corrosion-induced changes in phase composition and surface state occur. Thus, the separated-phase assessment is useful for identifying which phase and constituent element have the greatest initial tendency for metal ionization. As corrosion proceeds, however, selective dissolution may substantially alter the compositions and surface states of the individual phases, while interphase charge transfer may establish a common mixed potential. Consequently, subsequent corrosion behavior becomes difficult to predict quantitatively using the present fixed-composition framework. The present calculations do not explicitly include polarization kinetics, phase-area ratios, phase or grain boundaries, interphase galvanic coupling, or elastic and coherency interactions among neighboring phases. Accordingly, the calculated *E*_red,eq_ values should be interpreted as initial-stage thermodynamic screening descriptors rather than as direct predictions of corrosion rate, galvanic current, electrochemical mixed potential, or corrosion morphology. Actual molten-salt corrosion behavior may also be influenced by salt chemistry, impurity concentration, mass transport, and interfacial kinetics [40]. Nevertheless, the present results demonstrate that secondary-phase constitution and phase-specific redox stability can contribute significantly to the thermodynamic component of corrosion susceptibility in Ni-based alloys under molten-chloride conditions. The proposed framework therefore provides a thermodynamic basis for identifying susceptible secondary phases whose behavior cannot be inferred from nominal alloy composition or matrix properties alone.

## 4. Conclusions

CALPHAD-based phase prediction and representative-structure redox thermodynamics were combined to compare phase-specific susceptibility to metal ionization at 1000 K under a common idealized molten-chloride condition. The dominant Fe-rich α and γ phases in SA508 exhibited similar Fe-ionization potentials, indicating limited phase-to-phase variation. In IN625, the P phase exhibited the most negative potential, governed by Cr ionization, and therefore the highest thermodynamic susceptibility among the phases considered. In contrast, the Cr-rich carbide was less susceptible than the γ matrix, while the γ″ phase exhibited a less susceptible Nb-controlled response. These results show that the thermodynamic susceptibility of Ni-based alloys to metal ionization cannot be inferred solely from nominal alloy composition or matrix behavior; phase constitution, phase-specific composition, and the dominant ionizing element must instead be considered together. The calculated redox equilibrium potentials should be interpreted as thermodynamic descriptors rather than direct predictions of corrosion rate or electrochemical mixed potential. Nevertheless, the proposed framework provides a useful screening approach for identifying secondary phases with relatively high metal-ionization susceptibility in multicomponent alloys under molten-salt conditions.

## Figures and Tables

**Table 1:** Representative nominal chemical compositions of IN625 and SA508 selected within published composition limits [32,33] and used as inputs for the CALPHAD-based phase prediction (wt.%).

Elements (wt.%)	Fe	Cr	Ni	Mo	Nb	Al	Ti	C
SA508	Bal.	0.25	0.50	0.50	0.01	0.025	0.015	0.25
IN625	5.00	20.00	Bal.	8.00	4.15	0.40	0.40	0.10
**Elements (wt.%)**	**Mn**	**Si**	**Co**	**V**	**P**	**S**	**B**	**Ca**
SA508	1.50	0.40	-	0.05	0.025	0.025	0.003	0.015
IN625	0.50	0.50	1.00	-	-	-	-	-

## Data Availability

The original contributions presented in this study are included in the article. Further inquiries can be directed to the corresponding author.

## Abbreviations

The following abbreviations are used in this manuscript:

BCC	Body-centered cubic
CALPHAD	CALculation of PHAse Diagrams
FCC	Face-centered cubic
GGA	Generalized gradient approximation
IN625	Inconel 625
PFP	PreFerred Potential
SA508	SA508 low-alloy steel
TCP	Topologically close-packed

## References

[1] LeBlanc D. (2010). Molten salt reactors: A new beginning for an old idea. Nucl. Eng. Des..

[2] Serp J., Allibert M., Beneš O., Delpech S., Feynberg O., Ghetta V., Heuer D., Holcomb D., Ignatiev V., Kloosterman J.L. (2014). The molten salt reactor (MSR) in generation IV: Overview and perspectives. Prog. Nucl. Energy.

[3] Williams D.F. (2006). Assessment of Candidate Molten Salt Coolants for the NGNP/NHI Heat-Transfer Loop.

[4] DeVan J.H., Evans I.I.I. (1962). Corrosion Behavior of Reactor Materials in Fluoride Salt Mixtures.

[5] Delpech S., Cabet C., Slim C., Picard G.S. (2010). Molten fluorides for nuclear applications. Mater. Today.

[6] Guo S., Zhang J., Wu W., Zhou W. (2018). Corrosion in the molten fluoride and chloride salts and materials development for nuclear applications. Prog. Mater. Sci..

[7] Ding W., Bonk A., Bauer T. (2018). Corrosion behavior of metallic alloys in molten chloride salts for thermal energy storage in concentrated solar power plants: A review. Front. Chem. Sci. Eng..

[8] Sun H., Wang J., Li Z., Zhang P., Su X. (2018). Corrosion behavior of 316SS and Ni-based alloys in a ternary NaCl-KCl-MgCl_2_ molten salt. Sol. Energy.

[9] Sun H., Zhang P., Wang J. (2018). Effects of alloying elements on the corrosion behavior of Ni-based alloys in molten NaCl-KCl-MgCl_2_ salt at different temperatures. Corros. Sci..

[10] Mathew M.D., Parameswaran P., Rao K.B.S. (2008). Microstructural changes in alloy 625 during high temperature creep. Mater. Charact..

[11] Floreen S., Fuchs G.E., Yang W.J. (1994). The Metallurgy of Alloy 625. Superalloys.

[12] Keller T., Lindwall G., Ghosh S., Ma L., Lane B.M., Zhang F., Kattner U.R., Lass E.A., Heigel J.C., Idell Y. (2017). Application of Finite Element, Phase-field, and CALPHAD-based Methods to Additive Manufacturing of Ni-based Superalloys. Acta Mater..

[13] Gallmeyer T.G., Moorthy S., Kappes B.B., Mills M.J., Amin-Ahmadi B., Stebner A.P. (2020). Knowledge of process-structure-property relationships to engineer better heat treatments for laser powder bed fusion additive manufactured Inconel 718. Addit. Manuf..

[14] Suave L.M., Cormier J., Villechaise P., Soula A., Hervier Z., Bertheau D., Laigo J. (2014). Microstructural Evolutions During Thermal Aging of Alloy 625: Impact of Temperature and Forming Process. Met. Mater. Trans. A.

[15] Yu L.-J., Marquis E.A. (2019). Precipitation behavior of Alloy 625 and Alloy 625 plus. J. Alloys Compd..

[16] Lass E.A., Stoudt M.R., Katz M.B., Williams M.E. (2018). Precipitation and dissolution of δ and γ″ during heat treatment of a laser powder-bed fusion produced Ni-based superalloy. Scr. Mater..

[17] Baldan R., da Silva A.A.A.P., Tanno T.M., da Costa E.T., Brentegani J.V.N., Couto A.A. (2020). Experimental Investigation of Delta Phase Precipitation in Inconel 625 Superalloy Aged at 550, 625 and 725 °C. Mat. Res..

[18] Dubiel B., Sieniawski J. (2019). Precipitates in Additively Manufactured Inconel 625 Superalloy. Materials.

[19] Azadian S., Wei L.-Y., Warren R. (2004). Delta phase precipitation in Inconel 718. Mater. Charact..

[20] Sundararaman M., Mukhopadhyay P., Banerjee S. (1992). Some aspects of the precipitation of metastable intermetallic phases in INCONEL 718. Met. Trans. A.

[21] Lee J.-S., Lee Y., Kwon S.I., Shin J., Lee J.-H. (2021). Localized Corrosion Behavior of Inconel 718 in a Chloride-Containing Aqueous Solution. Corros. Sci. Technol..

[22] Alano J.H., Siqueira R.L., de Oliveira A.D., Vacchi G.D.S., Della Rovere C.A., Kuri S.E. (2020). Effect of TCP phase formation on the electrochemical corrosion behavior of the nickel-based superalloy UNS N26455. Corros. Sci..

[23] Pettit F.S., Meier G.H. (1984). Oxidation and Hot Corrosion of Superalloys. Superalloys.

[24] Cabrini M., Lorenzi S., Testa C., Brevi F., Biamino S., Fino P., Manfredi D., Marchese G., Calignano F., Pastore T. (2019). Microstructure and Selective Corrosion of Alloy 625 Obtained by Means of Laser Powder Bed Fusion. Materials.

[25] Lee U., Kim M.W., Na J., Lee M., Kim S.J., Kim D.-J., Yoon Y.S. (2025). A Study on the Corrosion Behavior of Fe/Ni-Based Structural Materials in Unpurified Molten Chloride Salt. Materials.

[26] Pillai R., Sloof W.G., Chyrkin A., Singheiser L., Quadakkers W.J. (2015). A new computational approach for modelling the microstructural evolution and residual lifetime assessment of MCrAlY coatings. Mater. High Temp..

[27] Pillai R., Raiman S.S., Pint B.A. (2021). First steps toward predicting corrosion behavior of structural materials in molten salts. J. Nucl. Mater..

[28] Pillai R., Sulejmanovic D., Lowe T., Raiman S.S., Pint B.A. (2023). Establishing a Design Strategy for Corrosion Resistant Structural Materials in Molten Salt Technologies. JOM.

[29] Savara A., Pillai R. (2024). Preliminary Assessment of Models for Generating Predictions of Long-Term Corrosion in Molten Salts.

[30] Xu Z., Guan B., Wei X., Lu J., Ding J., Wang W. (2022). High-temperature corrosion behavior of Inconel 625 alloy in a ternary molten salt of NaCl-CaCl_2_-MgCl_2_ in air and N_2_. Sol. Energy.

[31] Andersson J.-O., Helander T., Höglund L., Shi P., Sundman B. (2002). Thermo-Calc & DICTRA, computational tools for materials science. Calphad.

[32] Special Metals Corporation, INCONEL Alloy 625, Special Metals Corporation, Huntington, WV, n.d. https://www.specialmetals.com/documents/technical-bulletins/inconel/inconel-alloy-625.pdf.

[33] (2023). Standard Specification for Quenched and Tempered Vacuum-Treated Carbon and Alloy Steel Forgings for Pressure Vessels.

[34] Takamoto S., Shinagawa C., Motoki D., Nakago K., Li W., Kurata I., Watanabe T., Yayama Y., Iriguchi H., Asano Y. (2022). Towards universal neural network potential for material discovery applicable to arbitrary combination of 45 elements. Nat. Commun..

[35] Ham S., Kwon C., Kim M., Cha H.L., Kim H.-A., Yun J.-I., Park J.W., Lee J., Paek S., Zhang J. (2026). Corrosion-resistant coatings in molten salts suggested by computational phase-stability diagrams. Acta Mater..

[36] Gozlan E., Bamberger M., Dirnfeld S.F., Prinz B., Klodt J. (1991). Topologically close-packed precipitations and phase diagrams of Ni-Mo-Cr and Ni-Mo-Fe and of Ni-Mo-Fe with constant additions of chromium. Mater. Sci. Eng. A.

[37] Lei P., Zhou L., Zhang Y., Wang F., Li Q., Liu J., Xiang X., Wu H., Wang W., Wang F. (2024). Corrosion Behavior of Ni-Cr Alloys with Different Cr Contents in NaCl-KCl-MgCl_2_. Materials.

[38] Cwalina K.L., Demarest C.R., Gerard A.Y., Scully J.R. (2019). Revisiting the effects of molybdenum and tungsten alloying on corrosion behavior of nickel-chromium alloys in aqueous corrosion. Curr. Opin. Solid State Mater. Sci..

[39] Zhao W., Ni D., Qi W., Ding J., Lu J., Wang W., Wei X., Liu S. (2025). Corrosion mechanism of Cr-rich nickel-based alloys in a ternary molten salt: Morphology analysis and first-principles study of Cl adsorption. RSC Adv..

[40] Yu R., Gong Q., Shi H., Chai Y., Bonk A., Weisenburger A., Wang D., Müller G., Bauer T., Ding W. (2023). Corrosion behavior of Fe-Cr-Ni based alloys exposed to molten MgCl_2_-KCl-NaCl salt with over-added Mg corrosion inhibitor. Front. Chem. Sci. Eng..

